# Ghrelin regulating liver activity and its potential effects on liver fibrosis and *Echinococcosis*

**DOI:** 10.3389/fcimb.2023.1324134

**Published:** 2024-01-08

**Authors:** Jiang Zhu, Tanfang Zhou, Meng Menggen, Kalibixiati Aimulajiang, Hao Wen

**Affiliations:** ^1^ State Key Laboratory of Pathogenesis, Prevention and Treatment of High Incidence Diseases in Central Asia, Clinical Medicine Institute, The First Affiliated Hospital of Xinjiang Medical University, Urumqi, Xinjiang, China; ^2^ Department of Hepatobiliary and Hydatid Disease, Digestive and Vascular Surgery Center Therapy Center, The First Affiliated Hospital of Xinjiang Medical University, Urumqi, Xinjiang, China

**Keywords:** Ghrelin, liver, fibrosis, *Echinococcosis*, IGF - I

## Abstract

Ghrelin widely exists in the central nervous system and peripheral organs, and has biological activities such as maintaining energy homeostasis, regulating lipid metabolism, cell proliferation, immune response, gastrointestinal physiological activities, cognition, memory, circadian rhythm and reward effects. In many benign liver diseases, it may play a hepatoprotective role against steatosis, chronic inflammation, oxidative stress, mitochondrial dysfunction, endoplasmic reticulum stress and apoptosis, and improve liver cell autophagy and immune response to improve disease progression. However, the role of Ghrelin in liver *Echinococcosis* is currently unclear. This review systematically summarizes the molecular mechanisms by which Ghrelin regulates liver growth metabolism, immune-inflammation, fibrogenesis, proliferation and apoptosis, as well as its protective effects in liver fibrosis diseases, and further proposes the role of Ghrelin in liver *Echinococcosis* infection. During the infectious process, it may promote the parasitism and survival of parasites on the host by improving the immune-inflammatory microenvironment and fibrosis state, thereby accelerating disease progression. However, there is currently a lack of targeted *in vitro* and *in vivo* experimental evidence for this viewpoint.

## 1 Introduction

Ghrelin is an endogenous ligand of the growth hormone secretagogue receptor (GHSR) discovered in rat and human stomach in 1999, and it is now recognized as the third hormone that regulates growth hormone (GH) secretion except growth hormone releasing hormone (GHRH) and somatostatin. It is an acylated peptide containing 28 amino acids, and its N-terminal 10 amino acid sequence is highly conserved in mammals, suggesting the importance of Ghrelin in performing biological functions (Yanagi et al., 2018). Ghrelin and its receptor GHSR1a are widely expressed in central systems such as the hypothalamus, pituitary, cerebral cortex, and striatum, as well as peripheral organs such as the gastrointestinal tract, liver, pancreas, heart, thyroid, breast, adrenal gland, testis and ovary. The fundus of the stomach is the main secretion area of Ghrelin, which is secreted by X/A-like cells in rodents and by P/D1 cells in humans (Date et al., 2000). Ghrelin exists in two forms in mammals: octanoylated Ghrelin and non-octanoylated Ghrelin, the ratio of which is 2:1 in the stomach and 1:10 in the plasma (Hosoda et al., 2000). Octanoylated Ghrelin is catalyzed by O-acyltransferase (GOAT) in the cytoplasm, and a form of octanoylation with octanoic acid in serine 3 (Hosoda et al., 2000; Yang et al., 2008), which depends on GHSR1a in the central system and peripheral target organs, plays biological roles such as maintaining energy homeostasis, regulating lipid metabolism, cell proliferation, immune response, gastrointestinal physiological activities, cognition, memory, circadian rhythm and reward effects (Al Massadi et al., 2011; Müller et al., 2015). In the early days, it was believed that non-octanoylated Ghrelin had no activity, but it was found that it also has the effect of regulating glucose and lipid metabolism, but the regulatory mechanism and dependent receptors are still unclear (Heppner et al., 2014; Hopkins et al., 2017).

Ghrelin had been confirmed to be negatively correlated with insulin resistance and positively correlated with cachexia (Müller et al., 2010; Vergani et al., 2021), and it could improve ischemic injury, inflammatory damage and fibrosis formation, accelerate tissue repair and perform some other protective effects in diseases of target organs including the brain, heart, gastrointestinal tract, pancreas and kidney. There is also a complex regulatory relationship between Ghrelin and the liver. Ghrelin, which could improve the outcome of many benign liver diseases, especially liver fibrotic diseases, could play a significant protective role. However, liver *Echinococcosis*, as a chronic parasitic infectious disease that could cause liver fibrosis and necrosis, it is still unclear whether Ghrelin is involved in regulating the process and outcome of the disease. Therefore, this review systematically summarizes the molecular mechanisms by which Ghrelin regulates liver growth metabolism, immune-inflammation, fibrosis state, proliferation and apoptosis, as well as its protective effects in liver fibrotic diseases, and combined with the current research status, proposes that Ghrelin may be involved in regulating the disease process of liver *Echinococcosis*.

## 2 Biological functions of Ghrelin in the liver

The liver is an important central regulatory organ for metabolic function. Various liver diseases are accompanied by pathophysiological changes caused by liver metabolic dysfunction, including insulin resistance, chronic inflammation, oxidative stress, mitochondrial dysfunction, endoplasmic reticulum stress, apoptosis, autophagy abnormalities, etc (Alkhouri et al., 2011; Harmon et al., 2011; Ezquerro et al., 2016). Studies have found that Ghrelin can activate many interactive and crosstalk signaling pathways through the central nervous system and peripheral target organs to regulate the metabolic activities of the liver and counteract the “multiple hit effect” caused by liver metabolic dysfunction. It is worth noting that the regulatory mechanism of Ghrelin has two sides at the hypothalamic level and the liver level, and there are unclear mechanisms that need to be further studied.

### 2.1 Ghrelin-GH-IGF- I growth axis regulating liver activity

**Figure 1:**
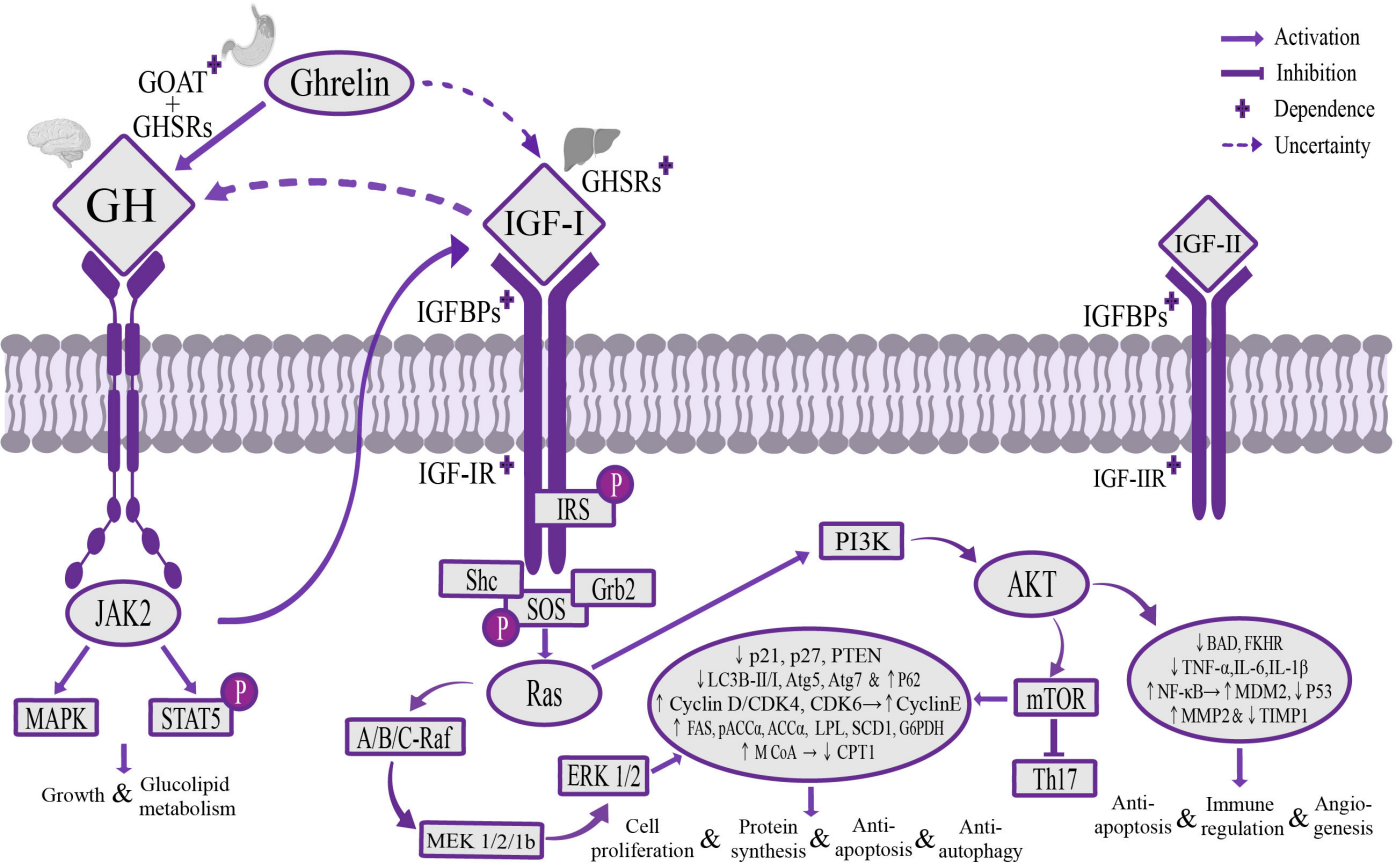
The mechanism of Ghrelin-GH-IGF-I growth axis regulating liver activity. Stomach-derived Ghrelin is transported to the hypothalamus through the blood circulation and afferent vagus nerve, where it’s catalyzed by GOAT to form octanoylated Ghrelin and then binds to GHSRs dependently to promote GH secretion. GH can regulate the downstream JAK2/STAT5 and MAPK signaling pathways to promote growth, maintain glycolipid homeostasis, and stimulate the secretion of IGF-I in the liver. IGF-I transports and activates IGF-IR through IGFBPs, regulates MEK/ERK 1/2 and PI3K/AKT/mTOR signaling pathways through IRS and Ras mediation, increases the expression of liposynthase, cyclin E and autophagy gene p62, inhibit the expression of lipolytic enzymes, anti-mitotic genes p21, p27, PTEN and autophagy-related genes Atg5 and Atg7, and reduce the ratio of autophagy marker LC3B-II/I, promote cell proliferation and protein synthesis, and resist apoptosis and autophagy, and can regulate the AKT signaling pathway to inhibit the expression of pro-apoptotic gene BAD and transcription factor FKHR, and up-regulate the expression of anti-apoptotic factor NF-кB and p53 key negative regulator MDM2, inhibit p53 expression and maintain dynamic balance of MMP2 and TIMP1, to play the role of promoting angiogenesis, reducing inflammatory injury, anti-apoptosis and regulating immune response. It’s worth noting that there is a potential direct regulatory pathway between Ghrelin and IGF-I, and a potential negative feedback regulatory pathway between GH and IGF-I. GOAT, o acyltransferase; GHSRs, growth hormone secretagogue receptors; IGFBPs, insulin-like growth factor binding proteins; IGF-IR, IGF-I receptor; MEK, mitogen-activated extracellular signal-regulated kinase; ERK 1/2, extracellular signal-regulated kinase 1/2; PI3K, phosphatidylinositol 3-kinase; AKT, serine/threonine kinase; mTOR, mammalian target of rapamycin; IRS, insulin receptor substrate; MMP2, matrix metalloproteinase 2; TIMP1, matrix metalloproteinase inhibitor 1.

The Ghrelin-GH-insulin-like growth factor-I (IGF-I) growth axis is the classic way for Ghrelin to regulate liver metabolic activity through the “gastrointestinal-brain-liver axis” (Hevrøy et al., 2011; Boguszewski et al., 2016; Wang et al., 2021). Under stress, such as energy homeostasis imbalance in the body, glucose-sensing neurons activate sympathetic nerves to mediate gastric Ghrelin secretion and transport it to the hypothalamus through blood circulation and the afferent vague nerve. At the level of the hypothalamus, Ghrelin is centrally regulated, and after being acylated by GOAT, it binds to GHSR1a to regulate GH secretion (Yanagi et al., 2018; Cornejo et al., 2021). GH mediates fat oxidation breakdown and hepatic gluconeogenesis, reduces insulin sensitivity of adipose tissue and liver to maintain energy homeostasis (Doycheva et al., 2022), and can activate Janus Kinase 2 (JAK2)/transcription factors STAT5 and the mitogen-activated protein kinase (MAPK) signaling pathway to play the role of promoting growth metabolism and maintaining glycolipid homeostasis (Lanning and Carter-Su, 2006). A deficiency of GH or STAT5 could cause major changes in fat distribution and mobilization, leading to the occurrence of acquired metabolic liver diseases (Barclay et al., 2011). At the liver level, the Ghrelin/GH signaling pathway can regulate the expression and activity of IGF-I in the liver (van der Velden et al., 2022), and IGF-I could reversely inhibit GH secretion and play an important role in balancing GH and insulin secretion (Fang et al., 2022), but the mechanism of this negative feedback is unclear. IGF-I secreted by the liver is mainly combined into the IGF/insulin-like growth factor binding protein-3 (IGFBP-3) complex in circulating blood to be transported, and relying on the IGF-I receptor (IGF-IR), it plays an important role in metabolic regulation (Adamek and Kasprzak, 2018). Ghrelin-GH-IGF-I growth axis can inhibit the expression and activity of the fat-degrading enzyme carnitine palmitoyl transferase 1 (CPT1) at the liver level (Theander-Carrillo et al., 2006), and promote the expression and activity of fat storage enzymes, including fatty acid synthase (FAS), phosphorylated acetyl coenzyme A carboxylase α (pACCα), non-phosphorylated acetyl coenzyme A carboxylase α (ACCα), lipoprotein lipase (LPL), stearoyl-CoA desaturase 1 (SCD1), glucose-6 phosphate dehydrogenase (G6PDH), 6 phosphate dehydrogenase (6PGDH) and malonyl coenzyme A (M CoA), and inhibit insulin secretion, increase glucose utilization in adipose tissue, to promote the synthesis of lipid substances in the liver (Shan and Yeo, 2011; Shimizu et al., 2022). This could explain why high expression of Ghrelin leads to obesity and acquired metabolic liver diseases. Inhibition of IGF-I in rats could reduce adipose tissue by more than 25% within 3 months (Boucher et al., 2016). In addition, the Ghrelin-GH-IGF-I growth axis, through the mediation of insulin receptor substrate (IRS) and rat sarcoma (Ras), could regulate Mitogen-activated extracellular signal-regulated kinase (MEK)/extracellular regulated proteinhnase l/2 (ERK1/2) (Kotta et al., 2022) and phosphatidylinositide 3-kinases (PI3K)/serine/threonine kinase AKT/mammalian target of rapamycin (mTOR) signaling pathway (Zhu et al., 2018; Zhang and Xie, 2020), up-regulating the expression of cyclin D and cyclin-dependent kinases CDK4, CDK6 and form cyclin D/CDK4 complex, promoting the release of transcription factor E2F through ELK1 in the Est transcription factor family, increasing the expression of cyclin E, up-regulating the expression of autophagy gene p62, and inhibiting the anti-mitotic genes p21, p27, phosphatase and tensin homolog (PTEN) and the expression of autophagy genes Atg5 and Atg7, as well as reducing the ratio of autophagy marker LC3B-II/I, to play a role in promoting cell proliferation and protein synthesis, and resisting apoptosis and abnormal autophagy (Sengupta and Henry, 2015; Firmenich et al., 2020). It could also regulate the AKT signaling pathway to inhibit the expression of pro-apoptotic protein BAD and transcription factor FKHR, up-regulate the expression of anti-apoptotic factor NF-кB and p53 key negative regulator MDM2 (Liu et al., 2014), and reduce the proliferation and activation of hepatic stellate cells (HSCs) for promoting the formation of blood vessels, reduce inflammatory damage, fibrosis formation, anti-apoptosis and regulation of immune response (Adamek and Kasprzak, 2018) (**Figure 1**).

Some studies had informed a conclusion that the FAS, ACCα and SCD1 of wild-type rats were significantly increasing by pumping Ghrelin into the ventricle, whereas they were significantly weakening under the intervention conditions of low-dose Ghrelin, GH deficiency and vagus nerve suppression. It indicated that the regulation of the Ghrelin-GH-IGF-I growth axis at the liver level may be Ghrelin dose-dependent, but also GH and vagus nervous system dependent (Theander-Carrillo et al., 2006). However, these conclusions have been challenged. Ghrelin levels could still increase by 40% after fasting in GH-deficient rats, and Ghrelin administration could significantly increase liver FAS, pACCα, ACCα, SCD1, G6PDH and 6PGDH levels to promote fat synthesis and up-regulate the expression and activity of AMP-activated protein kinase (AMPK) α1 and AMPK α2 to maintain the blood glucose homeostasis and inhibit plasma insulin levels (Sangiao-Alvarellos et al., 2009; Lee et al., 2022). Ghrelin administration in wild-type mouse hepatocyte culture *in vitro* also found that it can increase lipid accumulation (Li et al., 2014). In addition, clinical studies of GH deficiency also found that there is no correlation between the concentration of Ghrelin and the concentration of GH (Malik et al., 2004), suggesting that the Ghrelin-GH-IGF-I growth axis that regulates liver glucose and lipid metabolism may not depend on GH mediation. Similar studies further proposed that Ghrelin regulates liver glucose and lipid metabolism independently of the expression and activity of GH, cortisol and free fatty acids (FFA) (Vestergaard et al., 2017). Interestingly, Ghrelin administration did not cause significant changes in the expression and activity of CPT 1 and M CoA in the absence of GH, suggesting that the effect of regulating fat oxidation and decomposition may be GH-dependent (Sangiao-Alvarellos et al., 2009). It is worth noting that oral administration of Ghrelin receptor agonist MK-0677 could directly increase the secretion of IGF-I by 65%, indicating a close regulatory relationship between Ghrelin and IGF-1 (Campbell et al., 2018). Recent studies had pointed out that in the mice whose GOAT gene knockout blocked Ghrelin acylation, the plasma IGF-I decreased by 90% under starvation, and GH administration increased the phosphorylation level of STAT5, but failed to increase the serum IGF-I level or the plasma glucose level, and the plasma glucose level increased twofold after injection of IGF-1. It was suggested that there may be a direct regulatory pathway for the Ghrelin/IGF-I axis that is not entirely dependent on the GH/STAT5 signaling pathway (Boucher et al., 2016; Fang et al., 2022). Current studies have found that GHSR1a is expressed in rat vagus nerve sensory neurons and efferent neurons, and administration of Ghrelin to rats could significantly increase the activity of c-Fos protein, an activity marker of vagus nerve efferent neurons, and the activity of mTOR, suggesting that there may be a vagal direct regulatory pathway between Ghrelin and the liver (Kupari et al., 2019; Davis et al., 2020; Nagoya et al., 2020; Perelló et al., 2022). But the problem is that the neuroanatomical evidence is not enough to draw a firm conclusion to support the existence of GHSR1a in the terminal of vagal efferent (Cornejo et al., 2021). In addition, the indispensability of the vagus nervous system in the Ghrelin-GH-IGF-I growth axis is controversial, and administration of Ghrelin to rats with suppressed subdiaphragmatic vagal afferent could still stimulate acute feeding behavior (Arnold et al., 2006). However, studies using Ghrelin to stimulate feeding behavior as an evaluation standard were also controversial. Some studies have found that Ghrelin deficiency doesn’t affect feeding behavior, but only reduces susceptibility to diet-induced obesity (Li et al., 2012; McFarlane et al., 2014). How to maintain the dynamic balance between Ghrelin/IGF-I and GH cannot be well explained at present, and the research on the direct regulation pathway of Ghrelin/IGF-I may become one of the most interesting unsolved problems in the field of Ghrelin.

### 2.2 Ghrelin regulating liver activity through p53, AMPK, mTOR and NPY/AgRP signaling pathways

**Figure 2:**
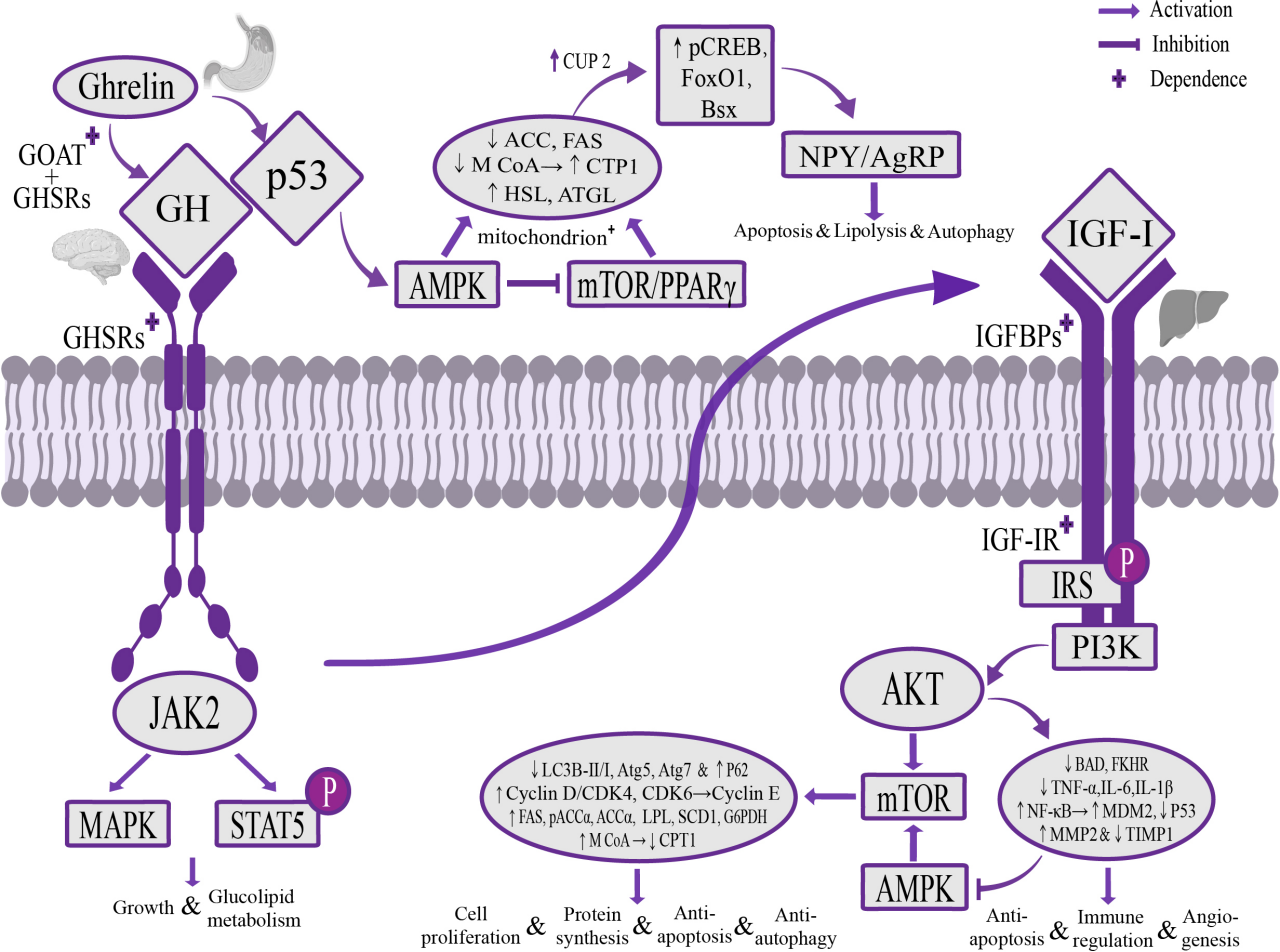
The mechanism of Ghrelin regulating liver activity through p53, AMPK, mTOR and NPY/AgRP signaling pathways. Stomach-derived Ghrelin is catalyzed by GOAT at the level of the hypothalamus to form octanoylated Ghrelin and then binds to GHSRs dependently, activates the AMPK signaling pathway through the Sirtuin1/p53 signaling pathway, inhibits the mTOR/PPARγ signaling pathway, reduces the secretion of liposynthetic enzymes ACC, FAS and M CoA, increases the secretion of lipolytic enzymes CPT1, HSL, ATGL and the activity of mitochondria, and up-regulates the expression of pCREB, FoxO1 and Bsx through UCP2, activates NPY/AgRP neurons, to play a role of stimulating food intake, promoting catabolism, apoptosis and autophagy. At the liver level, Ghrelin regulates the PI3K/AKT/mTOR and AMPK signaling pathways through the GH/IGF-I growth axis, increases the expression of liposynthetic enzymes, cyclin E and autophagy gene p62, and inhibits the expression of lipolytic enzymes and autophagy-related genes Atg5, Atg7, and the ratio of the autophagy marker LC3B-II/I, play a role in promoting cell proliferation and protein synthesis, anti-apoptosis and autophagy, and can regulate the AKT signaling pathway to inhibit the expression of pro-apoptotic gene BAD and transcription factor FKHR, as well as the expression of the anti-apoptotic factor NF-кB and the p53 key negative regulator MDM2, inhibit the expression of p53 and maintain the dynamic balance of MMP2 and TIMP1, play a role in promoting angiogenesis, reducing inflammatory damage, anti-apoptosis and modulating the immune response. AMPK, adenylate-dependent protein kinase; PPARγ, peroxisome proliferator-activated receptor γ; ACC, acetyl-CoA carboxylase; FAS, fatty acid synthase; M CoA, malonyl-CoA; CPT1, carnitine palmitoyl transferase 1; HSL, hormone-sensitive triglyceride lipase; ATGL, adipose triglyceride lipase; UCP2, uncoupling protein 2; pCREB, phosphorylated cyclic AMP response element binding protein; FoxO1, forkhead box protein O1; Bsx, brain-specific homologue protein homologue; MMP2, matrix metalloproteinase 2; TIMP1, matrix metalloproteinase inhibitor 1.

Tumor suppressor genes p53, AMPK, mTOR and **NPY**/AgRP are currently found to be metabolic sensors involved in the biological effects of Ghrelin (Hardie et al., 2012; Berkers et al., 2013; Quiñones et al., 2018; Liu et al., 2019). Unlike the Ghrelin-GH-IGF-I growth axis that regulates the activities of the liver, Ghrelin could independently regulate these signaling pathways in the central system to exert the opposite effect of the Ghrelin-GH-IGF-I growth axis. These interacting and crosstalking pathways work together to maintain the balance of liver metabolic activity. At the hypothalamic level, Ghrelin can regulate Sirtuin1 to deacetylate p53 (Puzio-Kuter, 2011; Velásquez et al., 2011), stimulate AMPK phosphorylation and inhibit mTOR/PPARγ signaling pathway (Budanov and Karin, 2008; Cariou et al., 2012; Li et al., 2014), thereby up-regulating the expression of lipoxygenase CPT1, hormone-sensitive lipase (HSL) and adipose triglyceride lipase (ATGL), inhibiting the expression of liposynthetic enzymes pACCα, ACCα, FAS, M CoA and transcription factor SREBP1, and increasing the expression of uncoupling protein 2 (UCP2) by changing the mitochondrial redox state, up-regulating the expression of phospho-cAMP response element binding protein (pCREB), forkhead box protein O1 (FoxO1), brain-specific homeobox protein homologue (Bsx), activate NPY/AgRP neurons (Kola et al., 2008; Dietrich et al., 2010; Quiñones et al., 2018), and jointly play a role in stimulating feeding and promoting effects of cellular catabolism, apoptosis and autophagy (López et al., 2008). At the liver level, the Ghrelin-GH-IGF-I growth axis up-regulates the expression of NF-κB and MDM2 through the AKT pathway, inhibits p53 activity against apoptosis, regulates the immune response (Liu et al., 2014), and down-regulates the expression of adiponectin receptor 2, inhibits AMPK phosphorylation (Kadowaki et al., 2006; Qin and Tian, 2010) and regulates the PI3K/AKT/mTOR signaling pathway to increase phosphorylation of downstream target S6 and transcription of glucose transporter 3 (GLUT3), promote glucose decomposition and uptake in liver tissue, and reduce mitochondrial oxidative phosphorylation, which can play a role in promoting cell anabolism, anti-apoptosis and autophagy (Cariou et al., 2012) (**Figure 2**).

Interestingly, among these mechanisms, administration of Ghrelin to mice that blocked the Sirtuin1/p53 signaling pathway (Velásquez et al., 2011) or knocked out hypothalamic CB 1 (Kola et al., 2013; Lim et al., 2013) failed to stimulate AMPK phosphorylation, but normal stimulation of GH secretion suggested that Ghrelin’s regulation of AMPK is dependent on the Sirtuin1/p53 signaling pathway and CB 1, and might be independent of the Ghrelin-GH-IGF-I growth axis. It had been confirmed that Ghrelin itself doesn’t have the effect of stimulating feeding, and its effect is mainly to activate the NPY/AgRP neurons in the arcuate nucleus of the hypothalamus. In mice with the absence of NPY/AgRP neurons (Verhulst et al., 2008) or the lack of upstream transcription factor Bsx (Sakkou et al., 2007), Ghrelin administration failed to stimulate feeding, and this regulatory relationship was also dependent on the Sirtuin1/p53 signaling pathway, and Ghrelin couldn’t activate NPY/AgRP neurons in p53 knockout mice, but did not affect the changes in glucose tolerance and insulin sensitivity, suggesting that this pathway was independent of the Ghrelin-GH-IGF-I growth axis to exert regulatory effects (Quiñones et al., 2018). It is worth noting that the use of rapamycin to inhibit mTOR, the key factor by which Ghrelin regulates liver activity, in the treatment of liver diseases might seriously hinder the proliferation of liver cells, and mTOR-independent intervention in Ghrelin signaling pathways may have better research value in the treatment of liver diseases.

### 2.3 Ghrelin regulating liver activity through autophagy, NF-κB signaling pathway and T cell immunity

Autophagy is a double-edged sword that can promote the catabolism of defective organelles and excess components to maintain cellular and energy homeostasis, but excessive autophagy can lead to type II programmed cell death (Shintani and Klionsky, 2004). At present, it has been found that various liver diseases are accompanied by hepatic autophagy disorders, and Ghrelin can dynamically regulate abnormal autophagy of hepatocytes to exert a hepatoprotective effect (Mao et al., 2015b; Ezquerro et al., 2019). At the level of the hypothalamus, Ghrelin can activate the Y1 and Y5 receptors of NPY neurons to promote autophagy (Aveleira et al., 2015; Ferreira-Marques et al., 2016). At the liver level, the Ghrelin-GH-IGF-I growth axis can regulate the downstream liver kinase B1 (LK B1)/AMPK and the P13K/AKT/mTOR signaling pathways, down-regulate the expression of Atg5, Atg7 and the expression of LC3B-II/I ratio, and up-regulate the expression of p62, to inhibit autophagy (Ezquerro et al., 2016; Zhang et al., 2018). It is controversial that autophagy is inhibited after GOAT, on which Ghrelin acetylation must depend, is blocked, but some studies have pointed out that it could activate the AMPK/mTOR signaling pathway to promote autophagy (Zhang Y. et al., 2015; Zhang et al., 2018), and clinical studies have found that increasing the expression of Ghrelin in old patients with chronic liver disease or liver injury could moderately induce autophagy and promote the repair of chronic liver disease and liver regeneration (Liu et al., 2018; Escobar et al., 2019; Bi et al., 2020; Xu et al., 2020). This might be due to the duality of Ghrelin in regulating autophagy at the hypothalamic level and the liver level. Ghrelin may have dual effects on dynamic homeostasis autophagy reduction and excessive autophagy. The study of Ghrelin’s role in regulating autophagy could provide some new insights for the development of drugs for the treatment of chronic liver diseases.

In addition, there are conflicting conclusions about the mechanism by which Ghrelin activates NF-κB at the liver level. In tumor diseases, the high expression of Ghrelin is generally believed to activate the NF-κB signaling pathway, inhibit the expression of p53, and cause tumor cell proliferation and migration (Chen et al., 2011; Tian et al., 2013). However, in the studies of liver steatosis, inflammatory injury and liver fibrosis, the high expression of Ghrelin was believed to inhibit the NF-κB/p65 signaling pathway by blocking the degradation of IκBα, thereby reducing the expression of tumor necrosis factor-α (TNF-α), interleukin (IL)-6, IL-8 and IL-1β, against lipotoxicity and inflammation (Hou et al., 2009; Ramachandran et al., 2012; Mao et al., 2015a), and Ghrelin could activate mTOR (Li et al., 2017) and regulate the expression of NOD2, an upstream intracellular receptor of NF-κB (Peng et al., 2012), to inhibit the expression of NF-κB. This might suggest that there were other regulatory pathways between Ghrelin and NF-κB, or that they were crosstalked by other regulatory pathways due to competition with p53 for common binding substances (Carrà et al., 2020).

There is often an interaction and crosstalk relationship between metabolism and immunity, and Ghrelin has also been proven to have the function of simultaneously regulating the body’s neuroendocrine and immune responses. T cells were found to contain the expression of Ghrelin and its receptors (Dixit et al., 2004). Studies had pointed out that Ghrelin could inhibit STAT3 phosphorylation and IL-17 secretion by regulating the mTOR signaling pathway, and regulate the differentiation of Th17 cells (Xu et al., 2015). In addition, Ghrelin had also been found to be involved in the process of suppressing Th1 type T cell immunity and promoting Th2 type and Tregs type T cell immunity against inflammation (Symonds et al., 2009; Yasen et al., 2021a). Therefore, Ghrelin was also considered an immune factor regulating immune homeostasis. In addition, Ghrelin could also increase the expression of B-cell lymphoma-2 (bcl-2) and endothelial nitric oxide synthase (eNOS), reduce the expression of TNF-α and liver tissue collagen level, anti-apoptosis and mediate NO release to play a hepatoprotective role (Kabil et al., 2014).

### 2.4 Effects of age and the circadian clock on the regulation of liver activity by Ghrelin

Studies had shown that aging is closely related to the Ghrelin-GH-IGF-I growth axis, and the circulating levels of Ghrelin and IGF-I decrease with age (Amitani et al., 2017). In aging liver cells, Ghrelin or IGF-I deficiency could activate p53, CDK4/6 inhibitory protein p16INK4a and CCAAT-enhancer binding protein α (C/EBPα), inhibit cell proliferation and promote cell Apoptosis and excessive autophagy (Zhao et al., 2022). Therefore, some scholars put forward the hypothesis that Ghrelin is anti-aging, but it’s controversial. Some studies had suggested that Ghrelin deficiency did not affect the lifespan of wild-type mice (Guillory et al., 2017), and pointed out that Ghrelin shows a tendency to increase with aging by inhibiting the formation of the C/EBPa-p300 complex, and promoting liver lipid accumulation and degeneration (Guillory et al., 2018). Ghrelin is also a regulator of circadian rhythm, which can participate in the regulation of circadian rhythm through the central system and target organs such as the liver and kidney (Reinke and Asher, 2016; Song and Rogulja, 2017; Saran et al., 2020). Studies had pointed out that high expression of Ghrelin could activate the hepatic mTOR/S6 signaling pathway in both *in vivo* and *in vitro* experiments, regulate the expression and rhythmicity of hepatic circadian locomotor output cycles kaput ear dichroism (Clock) and coding cycle Protein 2 (Per 2), restore circadian rhythm disturbance, improve hepatic steatosis, and reduce chronic inflammation (Wang et al., 2018a). In addition, the high expression of Ghrelin could activate the downstream AMPK signaling pathway (Lamia et al., 2009), mTOR signaling pathway (Kim et al., 2011), AKT signaling pathway (Luciano et al., 2018) and Sirtuin1/p53 signaling pathway (Zhou et al., 2014), by changing cryptochromes (Cry), the phosphorylation state of brain and muscle ARNT-like protein 1 (Bmal 1) and Clock improves the disorder of circadian rhythm, promotes protein synthesis and rhythmic detoxification.

## 3 Ghrelin and liver fibrosis diseases

Current studies have proposed that liver fibrosis is a “wound healing response”. After liver cell injury, it could inhibit the AMPK/mTOR signaling pathways and activate TGF-β1/Smad3 (Song et al., 2019), Sirtuin1/p53 (Song et al., 2019; Li et al., 2020) and NF-κB (Luedde and Schwabe, 2011) signaling pathways to promote autophagy and induce HSC proliferation and activation, destroy the dynamic balance of matrix metalloproteinase 2 (MMP2) and tissue inhibitor of matrix metalloproteinases 1 (TIMP1), causing excessive accumulation of type I and type III collagen-based extracellular matrix to regulate the progression of liver fibrosis. The key factors of the above signaling pathways have the potential to become key targets for improving liver injury and inflammation, as well as the progression of liver fibrosis. Ghrelin has been found to have a positive hepatoprotective effect in fatty liver disease and inflammatory liver disease, and may become a new drug target involved in the evaluation of liver reserve function and liver disease treatment in the future (Mao et al., 2015a; Ezquerro et al., 2020). Inhibition of the Ghrelin-GH-IGF-I growth axis could lead to the rapid progression of non-alcoholic fatty liver disease to liver cirrhosis (Doycheva et al., 2022). Many animal experiments had shown that high expression of Ghrelin can activate PI3k/Akt/mTOR (Moreno et al., 2010) and AMPK signaling pathways (Petrescu et al., 2020), inhibit TGF-β1/Smad3 (Mao et al., 2015b) and NF-κB/p65 (Mao et al., 2015a) signaling pathways, and effectively reduce secretion of TGF-β1, C-C motif ligand 2 (CCL2) and α-smooth muscle actin (α-SMA), restore the homeostasis of MMP2 and TIMP1, and reduce LC3B-II/I ratio, increase the expression of p62, to exert the effects of reducing oxidative stress and inflammatory injury of liver cells, resisting liver cell apoptosis and excessive autophagy, inhibiting HSC activation, and improving the progress of liver fibrosis (Petrescu et al., 2020). In the study of the progression of liver fibrosis induced by Ghrelin-deficient mice, liver fibrosis significantly worsened compared with wild-type mice, and the number of hepatic fibrotic cells was reduced by 25% after treatment with Ghrelin (Moreno et al., 2010). It should be noted that blocking the TGF-β1/Smad3 signaling pathway could inhibit the activation of Sirtuin1/p53, reduce HSC proliferation and activation, and improve the development of liver fibrosis. Ghrelin could inhibit the TGF-β1/Smad3 signaling pathway, but in the absence of Sirtuin1/p53 could not play a normal role (Velásquez et al., 2011; Porteiro et al., 2013). Their regulatory mechanism remains to be studied. Studies had also pointed out that high expression of Ghrelin could increase the expression of bcl-2 and eNOS in rats with liver fibrosis, and significantly reduce the levels of serum liver enzymes, TNF-α and liver tissue collagen, suggesting that Ghrelin could resist liver cell apoptosis and mediate NO release to fight liver fibrosis (Kabil et al., 2014). In addition, IGF-I was found to directly inactivate HSCs, improve portal pressure, bacterial translocation, endotoxemia, and collagen levels, reverse hepatic insulin resistance to protect liver cells from damage, stimulate the secretion of cell growth factors, and lead to liver regeneration (Sanz et al., 2005; Sobrevals et al., 2010; Chishima et al., 2017), but the serum IGF-I level was significantly reduced in cirrhotic patients (Blaas et al., 2010; Abdel-Wahab et al., 2015). Therefore, the IGF-I signaling pathway has the potential to diagnose and treat liver fibrosis, and is considered by many scholars to be a surrogate marker for evaluating liver reserve function.

Ghrelin in patients with non-alcoholic steatohepatitis (Yalniz et al., 2006), alcoholic hepatitis (Moreno et al., 2010), chronic hepatitis C (Kawaguchi et al., 2013; Hamdy et al., 2018) or chronic hepatitis B (Zhang X. et al., 2015) was significantly lower than that in healthy patients, after progressing to cirrhosis, it continued to decrease significantly, and it was significantly lower in patients with decompensated liver cirrhosis than in patients with compensated liver cirrhosis (Breidert et al., 2004; Kalaitzakis et al., 2007; Diz-Lois et al., 2009). The same conclusion was also found for cirrhosis caused by autoimmune liver disease in children (Dornelles et al., 2013). Some clinical studies had further proposed that Ghrelin could be considered a non-invasive diagnostic marker for patients with chronic liver disease to progress to cirrhosis, pointing out that serum Ghrelin levels below 850 pg/mL could be considered to progress to cirrhosis from chronic liver diseases, and those below 440 pg/mL, progression to decompensated cirrhosis might be considered (Elaghori et al., 2019). A clinical study of chronic hepatitis C pointed out that liver expression of the Ghrelin gene was related to the expression of the fibrosis gene. Ghrelin gene polymorphisms (-994CT and -604GA) effectively improved the progression of liver fibrosis, and used bile duct ligation to induce liver fibrosis in rats. During the formation process, 1534 genes hepatic expression was stimulated and 997 genes hepatic expression was inhibited. Ghrelin administration could improve hepatic expression of 231 genes, including type II collagen α1, fibrinogen activator-urokinase receptor, MMP2 and chemokine receptor 5 (Moreno et al., 2010). However, the conclusion that Ghrelin improved the development of liver fibrosis had given rise to different opinions in some clinical studies (**Table 1**). A clinical study found that the serum Ghrelin level of patients with fibrosis stage ≥ 2 was twice that of patients with fibrosis stage < 2 (Estep et al., 2011). Many clinical studies had also pointed out that serum Ghrelin level was positively correlated with the severity of patients with alcoholic cirrhosis, viral cirrhosis, primary biliary cirrhosis, and cirrhosis caused by hepatocellular carcinoma, and the level of Ghrelin in Child C stage cirrhosis was significantly higher than Child A/B stage patients (Tacke et al., 2003; Ataseven et al., 2006; Takahashi et al., 2006; El-Shehaby et al., 2010), the highest level was almost three times that of healthy patients, and the patients with ascites, upper gastrointestinal bleeding and hepatic encephalopathy were significantly higher than those without the above syndrome (Naguib et al., 2021). The reason might be that severe malnutrition and hypermetabolism accompany the progression of liver cirrhosis, and as an early indicator of malnutrition, the increase of Ghrelin reflects an adaptive compensatory mechanism, which is to activate the hypothalamus NPY/AgRP neurons and Ghrelin-GH-IGF-I growth axis by increasing the expression of Ghrelin, and thus stimulate feeding and maintain energy homeostasis (Elbadri et al., 2011; Quiñones et al., 2020). Another possible explanation is that liver cirrhosis is often accompanied by liver failure, cachexia, endotoxemia, and hemodynamic abnormalities. These changes might affect the levels of cytokines and vasoactive substances in the blood, including Ghrelin (Frascarelli et al., 2003). A third explanation was a hypothesis that micronutrient deficiencies or toxic products from protein breakdown in the progression of cirrhosis might impair appetite-regulating hypothalamic NPY/AgRP neurons, leading to altered Ghrelin sensitivity (El-Shehaby et al., 2010). The regulatory mechanism and biological effects between Ghrelin and liver fibrosis need to be further studied.

**Table 1:** Ghrelin expression in clinical studies related to liver fibrosis and liver cirrhosis.

Expression of Ghrelin (Low)		Expression of Ghrelin (High)	
Object of study	Ref.	Object of study	Ref.
liver cirrhosis	(Elaghori et al., 2019).	primary biliary cirrhosis	(Naguib et al., 2021).
liver cirrhosis	(Dornelles et al., 2013).	liver cirrhosis due to viral hepatitis C	(Elbadri et al., 2011).
liver fibrosis due to viral hepatitis C	(Moreno et al., 2010).	liver fibrosis due to NAFLD	(Estep et al., 2011).
liver failure requiring transplantation	(Diz-Lois et al., 2009).	liver cirrhosis due to viral hepatitis	(El-Shehaby et al., 2010).
liver cirrhosis	(Kalaitzakis et al., 2007).	liver cirrhosis due to viral hepatitis B	(Ataseven et al., 2006).
primary biliary cirrhosis	(Breidert et al., 2004).	liver cirrhosis	(Takahashi et al., 2006).
		liver cirrhosis due to chronic liver disease	(Tacke et al., 2003).

In the clinical studies of (Elaghori et al., 2019), (Dornelles et al., 2013), (Moreno et al., 2010), (Diz-Lois et al., 2009), (Kalaitzakis et al., 2007) and (Breidert et al., 2004), the serum Ghrelin level of patients was inversely related to the severity of liver fibrosis and liver cirrhosis. In the clinical studies of (Naguib et al., 2021), (Elbadri et al., 2011), (Estep et al., 2011), (El-Shehaby et al., 2010), (Ataseven et al., 2006), (Takahashi et al., 2006) and (Tacke et al., 2003), the serum Ghrelin level of patients was positive correlated with the severity of liver fibrosis and liver cirrhosis.

## 4 Ghrelin and liver *Echinococcosis*

Liver *Echinococcosis* is a parasitic infectious liver disease that causes liver fibrosis and necrosis and continues to grow slowly in the host body. It can cause inflammation and destroy the normal structure of liver tissue, endangering human health (Wen et al., 2019; Woolsey and Miller, 2021). Previous studies have suggested that innate immune pathways such as inflammatory vesicles and Toll-like receptor activation, and hepatocyte apoptosis are the host’s primary line of defense against the progression of liver *Echinococcosis* infection (Vuitton, 2003; Inclan-Rico and Siracusa, 2018; Bakhtiar et al., 2020). Recently, more and more studies have found that growth metabolic pathways are activated during the process of liver *Echinococcosis* infection and interact with the immune-inflammation and fibrosis pathways to jointly regulate the outcome of the disease (Seoane et al., 2016; Cheng et al., 2017; Liu et al., 2017; Yin et al., 2018; Wang et al., 2019; Lin et al., 2021; Yang et al., 2022). However, to date, few studies have addressed the various outcomes following interventions in growth metabolic pathways during the progression of liver *Echinococcosis*. In-depth studies of the impact of metabolic pathways on liver *Echinococcosis* may help further reveal the disease mechanisms of echinococcal infection.

Previous studies have pointed out that the imbalance of Th1/Th2 type cellular immunity is an important factor causing immune tolerance and immune evasion of the parasites in the liver *Echinococcosis*. In the early stage of echinococcal infection, the Th1-type cellular immune response is dominant, and in the advanced stage, the Th2-type cellular immune response is dominant (Gottstein et al., 1994; Emery et al., 1996; Emery et al., 1997; Bayraktar et al., 2005; Mezioug and Touil-Boukoffa, 2009; Mezioug and Touil-Boukoffa, 2012; Yasen et al., 2021b). And some studies have shown that during the process of liver *Echinococcosis* infection, Th1 type cellular immunity maintains a high response, which can exert a protective effect and reduce the damage to organs caused by parasitic infection, while Th2 type cellular immunity maintains a high response, which is beneficial to parasites development of immune tolerance and immune escape, which intensifies their parasitism and survival of the host (Ortona et al., 2003; Baz et al., 2006; Siracusano et al., 2012; Zhang et al., 2020; Jiménez et al., 2021; Zhang et al., 2021). The results of clinical studies also support this conclusion, pointing out that compared with healthy patients, patients with early infection and inactive *Echinococcosis* have higher expression of Th1-type cytokines in the serum and liver tissue, including IL-1β, 2, 15, 17, IFN-γ and TNF-α (Ma et al., 2014; Tian et al., 2020; Tilioua et al., 2020). Th2-type cytokines, including IL-4, 5, 6, 10 and 13, are highly expressed in the serum and liver tissue of patients with recurrent infection and active *Echinococcosis* (Mourglia-Ettlin et al., 2011; Ma et al., 2014; Tamarozzi et al., 2016; Yasen et al., 2021a). In addition, clinical studies have also pointed out that effective anti-infective treatment of *Echinococcosis* is positively correlated with maintaining a high response of Th1 type cellular immunity, while ineffective anti-infective treatment is more closely related to maintaining a high response of Th2 type cellular immunity (Siracusano et al., 2012; Gottstein et al., 2017). In addition, studies have shown that the NF- κB inflammatory signaling pathway (Tilioua et al., 2020; Lin et al., 2021) and the TGF-β1/Smad3 fibrosis signaling pathway (Wu et al., 2004; Banas et al., 2007; Liu et al., 2016; Tian et al., 2020) play a key role in regulating the immune-inflammation and fibrosis state of the host infected by *Echinococcosis*. Inhibition of these pathways could attenuate the host’s protective immune response and promote the disease progression of liver *Echinococcosis* infection. Clinical studies have shown that after echinococcus infects the host, it could activate the NF-κB and TGF-β1/Smad3 signaling pathways and increase the levels of proinflammatory factors IL-1β, IFN-γ, TNF-α, and IL-17 in the host’s serum and liver tissue, activate HSCs to mediate the secretion of profibrotic cytokines fibronectin, α-SMA and collagen I and III in liver tissue, and recruit inflammatory cells and tissue cells, including T cells, macrophages and fibroblasts/myofibroblasts in and around liver lesions, exert protective effects against parasitic infection (Grenard et al., 2001; Liu et al., 2003; Vuitton, 2003; Liu et al., 2006; Vuitton et al., 2006; Tilioua et al., 2020). In addition, TGF-β, as the main regulator of immune response, could induce and maintain T cell immunity and activate Th1-type immune response during the process of liver *Echinococcosis* infection to resist immune tolerance against parasites (Zhang et al., 2008; Feng et al., 2012; Wang et al., 2013; Ma et al., 2014; Liu et al., 2016; Tian et al., 2020). However, in patients with late chronic infection and relapse of *Echinococcosis*, the immune-inflammatory microenvironment shows weak expression (Wu et al., 2004; Banas et al., 2007; Tian et al., 2020; Tilioua et al., 2020), and the NF- κB and TGF-β1/Smad3 signaling pathways have interactions and crosstalk. The down-regulation of NF-κB also mediates the inhibition of the TGF-β1/Smad3 signaling pathway (Elsharkawy and Mann, 2007; Freudlsperger et al., 2013), which combined with the suppression of Th1-type cellular immunity, jointly promotes the progression of liver *Echinococcosis*.

**Figure 3:**
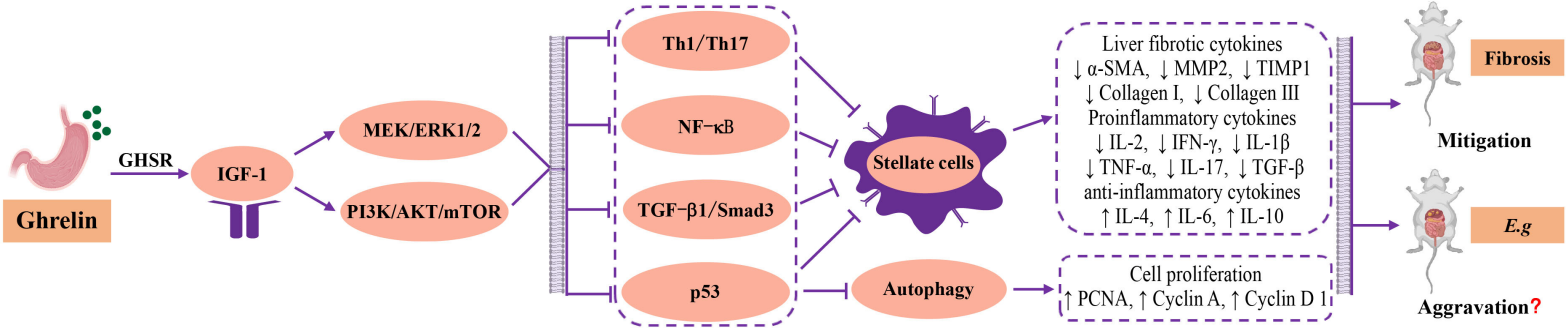
Regulatory mechanism and relationship among Ghrelin, liver fibrosis *and Echinococcosis*. The current research found that Ghrelin could stimulate the secretion of hepatic IGF-1 and regulate downstream MEK/ERK1/2 and PI3K/Akt/mTOR signaling pathways, and produced interactive inhibition of Th1 type cellular immunity, NF-κB inflammatory signalling pathway, TGF-β1/Smad3 fibrotic signalling pathway and p53 apoptotic factor, as well as synergistic inhibition of proliferation and activation of HSCs, downregulating the secretion of fibrotic cytokines a-SMA, MMP2, TIMP1, Collagen I and III, pro-inflammatory cytokines IL-2, IFN-g, IL-1b, TNF- a, IL-17 and TGF- b, and upregulating the secretion of the anti-inflammatory cytokines IL-4, IL-6, IL-10 and cell proliferation factors PCNA, Cyclin A, Cyclin D1, to improve the immune inflammation, fibrotic state, cell proliferation and repair in liver. The biological effects of Ghrelin could serve as a hepatic protective factor in improving disease outcomes of liver fibrosis. However, in liver Echinococcosis, the biological effect of Ghrelin may promote disease progression through the aforementioned regulatory mechanisms, but there is a lack of direct evidence. MEK, mitogen-activated extracellular signal-regulated kinase; ERK1/2, extracellular regulated proteinhnase l/2; PI3K, phosphatidylinositide 3-kinases; mTOR, mammalian target of rapamycin; TGF-β, Transforming growth factor-β; HSCs, hepatic stellate cells; a-SMA, a-smooth muscle actin; MMP2, matrix metallopeptidase 2; TIMP1, tissue inhibitor of matrix metalloproteinases 1; IL, interleukine; IFN-β, interferon-β; TNF-a, tumor necrosis factor-α; PCNA, proliferating cell nuclear antigen.

Studies have found that Ghrelin has anti-inflammatory effects that inhibit Th1 and Th17 immune responses and promote Th2 and Tregs T cell immunity (Dixit et al., 2004; Symonds et al., 2009; Stevanovic et al., 2012; Paoluzi et al., 2013; Xu et al., 2015). Current studies show that in the early and progressive stages of echinococcal infection, the JAK/STAT (Liu et al., 2017; Yang et al., 2022), MEK/ERK1/2 (Cheng et al., 2017; Lin et al., 2021) and PI3K/Akt/mTOR (Covarrubias et al., 2015; Seoane et al., 2016; Yin et al., 2018; Wang et al., 2019; Yang et al., 2022) signaling pathways are upregulated and may be involved in regulating the parasitism and survival of *Echinococcosis*. High expression of Ghrelin could significantly upregulate the MEK/ERK1/2 (Moreno et al., 2010; Kotta et al., 2022) and PI3K/Akt/mTOR (Zhu et al., 2018; Petrescu et al., 2020; Zhang and Xie, 2020) signaling pathways, and inhibit NF-κB (Zhou and Xue, 2009; Barazzoni et al., 2014; Mao et al., 2015b; Mao et al., 2015a) and TGF-β1/Smad3 (Mao et al., 2015b; Ezquerro et al., 2023) signaling pathways through interaction, significantly reducing the proinflammatory factors secreted by Th1-type cellular immunity, inhibiting the proliferation and activation of HSCs to restore the dynamic balance of MMP2 and TIMP1 and decrease the secretion of fibrotic cytokines α-SMA, collagen I and III, playing a role in reducing chronic inflammation and fibrosis formation in the liver. And activating the PI3K/Akt/mTOR signaling pathway could downregulate p53 to resist apoptosis, and studies have shown that p53 deficiency is more susceptible to parasitic infection (Kaushansky et al., 2013; Covarrubias et al., 2015; Gong et al., 2021). Although there is no direct evidence, the above-mentioned research conclusions suggest that Ghrelin has the potential to regulate the host’s immune inflammation, fibrosis formation and liver damage to mediate the progression of liver *Echinococcosis*. In addition, high expression of Ghrelin could activate IGF-1 through the “gastrointestinal-brain-liver axis”, and IGF-1 could directly inactivate HSCs to inhibit fibrosis formation (Sanz et al., 2005; Sobrevals et al., 2010; Chishima et al., 2017), and multiple parasite-related research results both pointed out that IGF-1 could promote parasitic parasitism and survival and accelerate disease progression *in vitro* and *in vivo* experiments (Vendrame et al., 2007; McDonald et al., 2014; Osorio et al., 2014; Ressurreição et al., 2016; de O Mendes-Aguiar et al., 2021). The authors’ research center also found that blocking IGF-1R could interfere with the glycolipid metabolism of echinococcus protoscoleces, causing vesicle collapse, and exerting an insecticidal effect against parasites (Li J et al., 2014). In addition, recent studies have found that Cyclin A, Cyclin D1, Cyclin E1 and PCNA are highly expressed in parallel in the early and progressive stages of liver *Echinococcosis* infection. The changes in Cyclin A, Cyclin D1 and PCNA are particularly significant and decrease in the later stages of infection (Zhang et al., 2012; Zhang et al., 2013). This enhancement of hepatocyte proliferation is believed to be beneficial to the repair of chronic liver injury. Ghrelin has been proven to significantly increase the secretion of the above cytokines and promote the proliferation and repair of liver cells (Lee et al., 2014; Wang et al., 2018b). However, it remains unclear whether changes in hepatocyte proliferation and repair status contribute to the progression of liver *Echinococcosis* (**Figure 3**).

There are currently no direct reports suggesting a correlation between Ghrelin and liver *Echinococcosis*. However, the cytokines and growth metabolic pathways regulated by Ghrelin have been proven to ameliorate host immune-inflammation, fibrosis formation and liver damage, and mediate the progression of liver *Echinococcosis* infection. Research on the intervention of Ghrelin to observe the progression of liver *Echinococcosis* will help reveal the pathogenic mechanism and new treatment mechanism of liver *Echinococcosis* from the growth metabolic pathway. Inhibiting Ghrelin may help improve the outcome of the disease, but this requires physical evidence from *in vitro* and *in vivo* experiments.

## 5 Conclusion

There is a close and complex regulatory relationship between Ghrelin and the liver. At present, Ghrelin’s indirect regulation of the liver through the “gastrointestinal-brain-liver axis” has been widely recognized. However, more and more studies have found that there are direct regulatory pathways between them, such as the vagal conduction pathway, but evidence is still lacking. In addition, Ghrelin can regulate immune homeostasis and fibrosis state, but how the interaction and crosstalk between them are generated remains to be studied in depth. In general, Ghrelin could inhibit the activation of immune-inflammation and fibrosis signaling pathways in liver fibrosis diseases to promote the proliferation and repair of liver cells, exert a protective effect, and improve disease progression. However, during the process of liver *Echinococcosis* infection, this protective effect may promote the parasitism and survival of parasites on the host and accelerate the progression of the disease. Inhibiting Ghrelin may help improve the outcome of liver *Echinococcosis*. However, this view currently lacks targeted experimental evidence *in vitro* and *in vivo*.

## Author contributions

JZ: Data curation, Resources, Validation, Writing – original draft, Writing – review & editing. TZ: Data curation, Formal analysis, Writing – review & editing. MM: Conceptualization, Methodology, Writing – review & editing. KA: Funding acquisition, Supervision, Visualization, Writing – review & editing. HW: Data curation, Methodology, Project administration, Supervision, Validation, Writing – review & editing.
